# Effectiveness of Video Self-Modeling in Teaching Unplugged Coding Skills to Children with Autism Spectrum Disorders †

**DOI:** 10.3390/bs15030272

**Published:** 2025-02-26

**Authors:** Erkan Kurnaz

**Affiliations:** Research Institute for Individuals with Disabilities, Anadolu University, 26470 Eskişehir, Türkiye; erkankurnaz@anadolu.edu.tr

**Keywords:** autism spectrum disorders, coding kills, unplugged coding skills, video self-modeling

## Abstract

This study examined the effectiveness of video self-modeling in teaching unplugged coding skills to children with autism spectrum disorder (ASD). The participants included one female and three male children with ASD, ages 10 to 12, in a multiple-probe design across subjects. The findings demonstrated that video self-modeling successfully facilitated the acquisition of unplugged coding skills for all four students. Additionally, all participants could generalize these skills to a new setting, and for those assessed, the skills were maintained for up to 12 weeks after the intervention. Social validity data collected from participants and their parents indicated positive perceptions of the approach. This study’s results highlight implications for instructional practices and future research.

## 1. Background

The education of children diagnosed with autism spectrum disorder (ASD) presents unique challenges that require a deep understanding of the disorder’s complexities (Zablotsky et al., 2015). These challenges emphasize the importance of developing learning processes tailored to their needs (Kamaruzaman et al., 2023). One of the critical aspects of this educational approach is involving children with ASD in the design of learning technologies. The active participation of these children ensures that the tools created are effective and inclusive (Hawas & Qasim, 2022). The perspectives of adult co-designers who work closely with children with ASD play an invaluable role in this process (Boyle & Arnedillo-Sánchez, 2022). These experts provide insights that help refine and adapt educational tools, ensuring that the learning approaches cater specifically to the needs of children with ASD. This involvement benefits the design of the tools and ensures that children with ASD become active participants in their learning journeys, enhancing both engagement and educational outcomes (Rogers & Dawson, 2010).

Transitioning from these specific needs in the education of children with ASD, it is essential to consider how broader educational trends, such as STEM, can be adapted to suit these unique requirements (Van Bergeijk et al., 2008). STEM education, an integrated approach to learning that combines Science, Technology, Engineering, and Mathematics, is increasingly recognized as vital for equipping students with essential 21st-century skills (Hsu & Tsai, 2022). Among the various components of STEM, robotic coding stands out as a critical element (Barker & Ansorge, 2007). It encompasses programming robots and integrates engineering, mathematics, and technology principles into a dynamic, hands-on learning experience (Silva et al., 2021). This method of education engages students in practical problem-solving, encourages critical thinking, and fosters creative design skills, leading to a more profound understanding of scientific and mathematical concepts (Sullivan & Bers, 2018).

Robotic coding develops technical programming skills and teaches students how to apply these skills in real-world scenarios, making learning more tangible and relevant (Silva et al., 2021). The National Science Foundation’s focus on learner-centered STEM methods, as outlined in the “Shaping the Future” report, emphasizes the importance of these educational approaches in developing skills and competencies necessary for modern life (Koculu et al., 2022). Moreover, holistic STEM education has enhanced students’ problem-solving skills, underlining its practical benefits (Kurt & Benzer, 2020). Studies have indicated that STEM education, mainly through robotics projects, promotes the development of crucial 21st-century skills such as creativity, critical thinking, and teamwork (Hanipah et al., 2019; Diquito et al., 2022). Given STEM’s structured and logical nature, particularly robotic coding, this approach seems especially suitable for children with ASD, who often thrive in structured environments (Bekele et al., 2013).

Robotic coding is particularly well-suited for children with ASD, as it aligns with their natural preference for structured, orderly environments (Knight et al., 2019a). This form of coding requires step-by-step reasoning and adherence to specific rules, which resonates with the structured perspective often preferred by these children. Block-based programming languages such as Scratch or Blockly enhance visual thinking by using colorful, drag-and-drop blocks to represent different coding commands (Resnick et al., 2009). This visual approach to coding makes the programming process more tangible and less abstract, aiding children with ASD in developing visual-spatial skills as they observe how their actions influence robot movements and responses (Hourcade et al., 2013). Moreover, the collaborative aspect of robotic coding projects is beneficial for improving social skills, as it encourages children to take turns, cooperate, and communicate effectively (De Korte et al., 2020). This environment provides a structured yet flexible setting for practicing social interactions, essential for children with ASD.

While robotic coding offers a more modern, technology-driven approach to learning, unplugged coding presents an alternative that can be equally beneficial, especially in the early stages of education (Brackmann et al., 2017). Unplugged coding refers to teaching coding and computational thinking concepts without reliance on computers or electronic devices. This method is particularly effective for tangibly and interactively introducing foundational coding principles to young learners. Unplugged coding activities often utilize physical objects, such as cards, puzzles, or manipulatives, to represent programming concepts and algorithms (Smith, 2018). This approach promotes computational thinking skills, problem-solving abilities, and logical reasoning in learners—particularly in the early stages of their education—and ensures inclusivity for diverse learners, including those with limited access to technology.

Unplugged coding activities can be incorporated into various educational settings, including elementary and middle school classrooms and informal learning environments such as after-school programs and community workshops (Sigayret et al., 2022). By engaging in hands-on, unplugged coding activities, students can develop a solid understanding of fundamental programming concepts, such as sequencing, loops, conditionals, and algorithms, before transitioning to computer-based coding environments (Kim et al., 2022). Educators and researchers have emphasized the importance of integrating unplugged coding into curricula to lay a strong foundation for computer science education and foster essential 21st-century skills in students (Bertacchini et al., 2022). This hands-on and interactive approach to unplugged coding is particularly advantageous for children with ASD, who may benefit from these activities’ tactile and visual aspects (Leach & Duffy, 2009).

The method of unplugged coding is particularly beneficial for children on the ASD spectrum as it aligns with their unique learning styles (Merino-Armero et al., 2022). The tactile and visual nature of unplugged coding activities helps develop computational thinking more concretely and tangibly, which is crucial for children with ASD who may find abstract concepts challenging (Merino-Armero et al., 2022; Dan & Changun, 2018). Completing tasks provides immediate, tangible feedback, fostering a sense of accomplishment and confidence in these children (Capecchi et al., 2022). Unplugged coding activities, which often involve physical and visual elements, make the principles of computer science more understandable and relatable for children on the ASD spectrum (McDaniel et al., 2020). For instance, using small, colored physical items, such as pegs and balls, in coding activities can deliver engaging and interactive learning experiences that are accessible and appealing to these children (Threekunprapa & Yasri, 2020). This approach enhances their understanding of computational concepts and contributes to cognitive development.

Furthermore, the immediate feedback from these activities is crucial for maintaining engagement and motivation in the learning process (Capecchi et al., 2022). Completing a task in unplugged coding can be immensely satisfying for children with ASD, helping to build their self-esteem and confidence in their abilities (Tsortanidou et al., 2023). This sense of achievement is essential for encouraging continued learning and exploration in computer science (Song, 2019).

Research on the instruction of fundamental coding skills for students with ASD and developmental disabilities is relatively limited. M. Taylor et al. (2017) sought to impart pre-coding skills to students with Down syndrome through the use of prompts and direct instruction. The results indicated that only one participant was able to retain these skills following the conclusion of the study. In a subsequent investigation, M. S. Taylor (2018) employed a methodology akin to that utilized with individuals with intellectual disabilities to teach coding skills. Although participants successfully navigated the learning phases, they encountered difficulties in generalizing these skills across varied contexts. Building upon these findings, Knight et al. (2019b) adopted a Model-Lead-Test (MLT) framework to instruct a student with ASD and severe behavioral issues in robotics coding. Their findings revealed a functional relationship between the employed instructional method and skill acquisition, as the student was able to maintain and generalize these skills to new coding tasks. Furthermore, Wright et al. (2019) conducted a study involving high school students with ASD, wherein they implemented digital, block-based coding. Their results demonstrated that participants not only acquired coding skills but also successfully generalized and self-generated innovative codes, underscoring the significance of structured, explicit instruction in the realm of STEM education.

The effectiveness of video-based modeling as an instructional method for teaching coding skills has been examined. Wright et al. (2019) evaluated video prompting to teach robotics and coding to middle school students with ASD. Their study confirmed that video-based modeling is an effective approach for improving coding accuracy and enabling students to transfer their knowledge to new tasks. Furthermore, they assessed the feasibility of the intervention in a public school setting, where a special education teacher successfully implemented the strategy. Additionally, Wright et al. (2020) conducted a systematic review of video modeling in STEM education. They found substantial evidence supporting video modeling in mathematics instruction but indicated a need for further research in other STEM disciplines. Moreover, Knight et al. (2019b) and Sola-Özgüç and Altın (2022) demonstrated the effectiveness of explicit instruction for teaching coding to students with ASD. Sola-Özgüç and Altın (2022) focused on a 10-year-old child with ASD and reported positive outcomes using structured, explicit teaching. These studies emphasize that children with ASD can acquire coding skills through various instructional methods, such as video-based modeling, explicit instruction, and hands-on robotics activities, highlighting the necessity of developing targeted educational programs to support their learning.

Video self-modeling (VSM) has emerged as a powerful tool, particularly tailored for the educational needs of students with ASD (Fragale, 2014). VSM utilizes edited video clips in which individuals view themselves successfully performing tasks, effectively addressing social and behavioral challenges in children with ASD (Diorio et al., 2018; Fuentes, 2016). This technique has been established as a potent intervention, significantly improving various skills and behaviors (Delano, 2007; Burton et al., 2013; McGuinty et al., 2018).

Research has consistently demonstrated the positive impact of VSM on social communication, language development, and behavior modification (Kabashi & Kaczmarek, 2017; Marcus & Wilder, 2009; Kabashi & Epstein, 2017). Bellini et al. (2007) identified that VSM significantly improved social engagement among young children with ASD by enhancing their participation in social interactions and peer communication. Similarly, Buggey et al. (1999) indicated that VSM effectively trained responsive behaviors, thereby improving social initiations in children with ASD. Boudreau and Harvey (2013) further illustrated how VSM increased recreational initiations, enabling children with ASD to engage more effectively in leisure activities. Moreover, VSM has been utilized to enhance conversational speech among children with ASD. Charlop and Milstein (1989) successfully employed video modeling techniques to teach conversational skills, resulting in significant improvements in verbal exchanges. Research also suggests that children with ASD frequently express a preference for video modeling over in vivo modeling. Geiger et al. (2010) investigated this preference and revealed that students with ASD respond more favorably to video-based interventions than to direct human modeling. Furthermore, VSM has proven to be an effective tool for developing academic skills in students with ASD. Burton et al. (2013) effectively utilized VSM to teach functional math skills, demonstrating its efficacy in breaking down complex tasks into manageable steps. Likewise, Decker and Buggey (2012) discovered that VSM facilitated reading fluency in children with learning disabilities, highlighting its broad applicability in educational contexts. Hart and Whalon (2012) extended these findings by employing iPads to implement VSM-based interventions, which increased academic engagement and response rates among adolescents with ASD and intellectual disabilities. Lang et al. (2009) explored the potential of VSM in teaching classroom rules and behavioral expectations to students with Asperger’s syndrome. Their study concluded that students not only learned and retained these rules over time but also generalized their learned behaviors across various contexts. Additionally, Williamson et al. (2013) validated the prerequisite skills necessary for VSM interventions, further broadening its applicability across different academic domains. Beyond academics, VSM has effectively been used to teach functional and adaptive behaviors to students with ASD. Wert and Neisworth (2003) demonstrated that VSM improved spontaneous requesting in children with autism, thereby enhancing their self-advocacy skills. Furthermore, Nikopoulos and Panagiotopoulou (2015) found that VSM was successful in reducing vocal stereotypy, contributing to the overall regulation of behavior within classroom environments. Additionally, Cihak and Schrader (2008) compared video self-modeling with video adult modeling and found that students with ASD were more effective in acquiring and maintaining tasks when observing themselves rather than an adult model. Their findings support the idea that personalized video interventions enhance learning and engagement in children with ASD. 

The versatility of VSM as both an instructional and behavioral intervention renders it an invaluable resource for students with ASD. Research indicates that VSM is effective in enhancing social engagement and communication (Bellini et al., 2007; Charlop & Milstein, 1989), as well as in improving academic performance and functional skills (Burton et al., 2013; Decker & Buggey, 2012). In conclusion, the literature provides robust support for the efficacy of VSM as an intervention strategy for individuals with ASD. It has successfully taught diverse social, communication, and functional competencies, highlighting its potential as a preferred method for instructing children with ASD. The aggregate evidence from various studies emphasizes VSM’s effectiveness in teaching a broad spectrum of skills, encouraging social initiation, and fostering independence among individuals with ASD.

However, there is a lack of research examining the influence of VSM on the instruction of unplugged coding skills as STEM skills in children with ASD. Therefore, this study aimed to investigate the effectiveness of VSM in teaching unplugged coding skills to children with ASD.

The following research questions were addressed:

1. Is applying the VSM technique effective for teaching unplugged coding skills to four children diagnosed with ASD?
2. Can these individuals demonstrate the acquired skills in maintenance and generalization?
3. Does the intervention have considerable social validity?

## 2. Method

### 2.1. Participants

This study involved one Turkish girl and three Turkish boys, aged 10 to 12, all diagnosed with ASD. Parental consent was secured for each participant. They attended a public school and participated in a private rehabilitation and special education program for one hour, twice a week after school. Participants were chosen based on the following inclusion criteria:

(a) Having a formal diagnosis of ASD: Participants’ diagnosis of ASD was verified by interviewing their parents and requesting a medical report.
(b) Ability to follow verbal instructions: Participants were assessed in an independent session by giving basic instructions such as “come”, “get”, “sit”, “get up”, and “look”. It was observed whether they followed the instructions or not.
(c) Ability to attend to visual and auditory stimuli for at least five minutes: In an independent session, participants were shown a video and listened to animal sounds via a tablet computer to assess this criterion. Participants’ attention spans were observed.
(d) Ability to imitate gross and fine motor skills: Participants were assessed for their ability to imitate gross and fine motor skills such as pointing, making a fist, standing up, and tapping their knees with the instruction “do this”.
(e) The ability to understand direction concepts when asked: The assessment was made using an A4-sized paper with arrows pointing in the “up”, “down”, “right”, and “left” directions. Participants were expected to show the desired aspects.
(f) Being comfortable with being videotaped and watching their videos: Participants were recorded in short videos in different settings, which were shown to them. This criterion was evaluated by observing their reactions while watching the videos.

All participants were diagnosed with ASD by child psychiatrists at a Turkish hospital. The Gilliam Autism Rating Scale-2 Turkish Version (GARS-2-TV; Diken et al., 2012) was used to verify these diagnoses. According to the GARS-2-TV, a score of 85 or higher indicates a high likelihood of ASD, scores between 70 and 84 suggest a potential ASD diagnosis, while scores of 69 or lower imply that ASD is unlikely. Table 1 presents the characteristics of the participants, and pseudonyms were assigned to ensure their privacy.

### 2.2. Experimental Design and Procedure

The effect of VSM on the development of unplugged coding skills in children with ASD was examined using a multiple-probe design across subjects, as described by Kazdin (2011). The research methodology encompassed several phases: the initial baseline, a second baseline following video session creation, intervention (instruction) sessions, and subsequent maintenance and generalization sessions, in line with the frameworks proposed by Goh and Bambara (2013) and Kurnaz and Yanardağ (2018). The primary variable measured in this research was the percentage of steps correctly executed in the coding worksheet skills. This study was conducted in accordance with the Declaration of Helsinki, and the protocol was approved by the Ethics Committee of Anadolu University. (Project identification code: 199400 and the date of approval: 30 November 2021).

***Probe Sessions.*** This study incorporated baseline probe sessions conducted as the first and second baselines. The objective of the initial baseline probe was to assess the participants’ independent skill level, with three sessions held over consecutive days. The second baseline probe aimed to identify any incidental learning during the video clip production process, as Goh and Bambara (2013) and Kurnaz and Yanardağ (2018) noted. A single opportunity procedure was employed during baseline and daily probe sessions. This procedure involved the following: (a) providing a verbal cue to capture the participant’s attention, (b) instructing the participant to perform the skill, (c) allowing a five-second window for skill initiation, (d) recording a correct behavior as a plus (+) for the target skill, and (e) noting an incorrect response as a minus (−), followed by terminating the assessment, as per Brown and Snell (2000). After each probe session, praise was given, irrespective of the participant’s performance. Baseline probe sessions lasted an average of 8 min for each subject (range: 7 min–9 min 30 s).

This study conducted daily and complete probe sessions in the same way as the baseline sessions. The daily probe session was designed to evaluate the participant’s current performance level and determine if it was appropriate to conclude the intervention process. These sessions were essential for monitoring the achievement of the target skill’s criterion. Each daily probe session, performed as a single trial after the intervention session, continued until a participant consistently displayed 100% accuracy in the target skills over three consecutive daily probe sessions.

Full probe sessions were scheduled at two critical junctures: before the commencement of the intervention and after the criterion for the target skill was met, as established by the outcomes of the daily probe sessions. These sessions were designed to continue until a stable response pattern was observed over a minimum of three consecutive sessions. The full probe sessions concluded with collecting comprehensive probe data from the final participant. This approach ensured a thorough and systematic evaluation of the intervention’s effectiveness in enhancing the target skills. The full probe sessions lasted an average of 27 min for each subject (range: 3 min–11 min).

***Making Video Sessions.*** After the initial baseline sessions, VSM clips were created. To accomplish this, video footage was captured showing the steps of the skill being demonstrated to all participants. The process steps specified in Akmanoğlu and Kurnaz (2014) were followed in preparing the videos. The videos were recorded at 18–135 mm speed using a Canon 7D Mark II DSLR camera on a tripod and an iPhone 12 Pro Max mounted on a DJI Gimbal. These sessions involved researchers, participants, and ancillary staff.

The ancillary staff demonstrated the target skill steps to the participants and occasionally provided partial physical prompts to assist participants in performing specific skill steps during the video recordings. The sessions incorporated complex step sequences and varied recording angles to mitigate the risk of incidental learning. A video camera captured each step and positioned the phone at different angles. These recordings were then edited to create the VSM clips for use in the intervention phase of the research.

The validity of the VSM clips was assessed by two special education experts, who confirmed that they were appropriate and understandable for the intervention. On average, recording sessions for each participant lasted around 25 h. According to Keenan and Nikopoulos (2006), VSM clips used in intervention sessions should not exceed five minutes. In this study, the average clip length was 2 min and 16 s, ranging from 1 min and 10 s to 3 min and 35 s. During each teaching session, participants viewed a complete sequence of the target behaviors once.

***Intervention Sessions*.** The VSM process for the unplugged coding skill was initiated when steady-state data were obtained after the second baseline probe session. The intervention sessions were conducted as follows: (a) The researcher gave the participant an attention cue (“Are you ready to watch a video clip?”) to focus on the task. If the participant responded verbally or with a gesture, the researcher gave verbal praise (“Great”). (b) The researcher and the participant sat side by side at a table, and the participant watched the self-modeling video clip on a tablet. After watching the video clip, the researcher asked, “The video is over. Do you want to watch it again?” and waited five seconds. If the participant wanted to watch the video clip again, the researcher provided the opportunity to watch it two more times. While watching the video clip, the researcher did not give the participant any information about the images. When the participant turned their back to the screen, the researcher gave a verbal warning (“Watch the video”) to maintain attention while watching the video clip. The researcher gave verbal praise when the participant watched the video clip (“Well done”). (c) Immediately after watching the VSM clip, the researcher and the participant moved to another area for the coding activity. (d) The researcher gave the participant an attention cue (“Are you ready to code?”) to focus on the task. (e) The researcher instructed the task when the participant responded verbally or with a sign (“Code”). The researcher gave verbal reinforcement (“Well done, bravo”) when the participant correctly performed the steps of the fiche-free coding skill. The intervention session ended when a participant gave an incorrect or no response for five seconds. Two trials were conducted for the target skill in each session, with a one-hour break between them. Intervention sessions lasted on average 3 h 32 min for each participant (range: 2 h 15 min–4 h 30 min).

***Maintenance and generalization sessions.*** Maintenance and generalization sessions were integral components of this study following the participants’ achievement of the instructional criterion, defined as the independent performance of the target skill. Maintenance sessions were scheduled one, two, and four weeks after the participants met the instructional criterion. The format and methodology of the maintenance sessions mirrored those of the probe sessions. The purpose of these sessions was to assess the durability of the skill acquisition over time, ensuring that the participants retained their ability to perform the target skill independently.

The generalization of skills across different settings was evaluated using a pre–post-test design. This involved conducting a probe session before the intervention process and another after the participants had achieved the unplugged coding skill criterion. The aim was to determine whether the skills learned in the intervention setting could be effectively transferred and applied in different environments or contexts. This aspect of the study was crucial for understanding the real-world applicability and effectiveness of the intervention.

### 2.3. Settings and Materials

The author carried out all instructional sessions, probes, maintenance, and generalization activities in a research laboratory at Anadolu University’s Research Institute for Individuals with Disabilities in Eskişehir, Türkiye. These one-on-one sessions took place five times a week in a laboratory that is 14 m long and 10 m wide, equipped with an acoustic lining.

A digital camera (Canon 7D Mark II DSLR) and an iPhone 12 Pro Max mounted on a DJI Gimbal were used to record each participant’s target behaviors. The videos and audio were processed using Adobe Premiere Pro 23.3, Adobe Media Encoder 23.2, and Adobe Audition 2022. iPads were used in this study to code the pages of the VEX 123 Robotics Worksheets used by the participants. Finally, a USB memory device, data collection forms, and a pencil were used to collect data.

The author documented all sessions utilizing a handheld digital video camera, in addition to any available cameras present within the environment. Subsequent to the experimental phase, a research assistant possessing a master’s degree and currently pursuing a doctorate meticulously reviewed the video recordings and gathered data to assess interobserver agreement. The author facilitated all study sessions, seated alongside participants throughout both the probe and video modeling phases. The video model was delivered via a tablet, ensuring that each session consisted solely of the author and a single participant. Generalization sessions were conducted in a distinct classroom within the same building, employing materials such as the coding worksheets utilized during this study. 

Research indicates that VSM is more beneficial than alternative video modeling methods (Buggey, 2005a); consequently, Noldus FaceReader software was employed to analyze participants’ emotional responses while viewing their videos, thereby supporting this assertion. Noldus FaceReader represents a sophisticated software application engineered to interpret facial expressions (Loijens & Krips, 2018; Noldus Information Technology, 2012). It utilizes advanced algorithms to automatically detect and analyze a diverse array of facial expressions, thereby providing insights into an individual’s emotional state. This technology, grounded in the principles of emotion psychology and computer vision, has been developed to recognize and quantify subtle variations in facial expressions, which signify various emotional conditions (Noldus Information Technology, 2012). The Noldus FaceReader is equipped with a comprehensive database of facial expressions, enabling it to interpret emotions across different individuals and contexts accurately. Its ability to provide objective, real-time analysis of facial expressions makes it an invaluable tool in research fields ranging from psychology and marketing to human-computer interaction and autism spectrum studies (Loijens & Krips, 2018). The software’s precision and versatility in detecting and interpreting emotional states underscore its significance in academic research and practical applications.

To determine if participants were satisfied with viewing their images—a prerequisite for applying video self-modeling—their reactions were analyzed while watching a 1-min video composed of their pictures and a 1-minute blank screen. The results of the FaceReader analysis of the participants are shown in Table 2. Statistical analyses revealed distinct emotional reactions (Neutral, Happy, Sad, Angry, Surprised, Frightened, Disgusted, Mood State, and Arousal) between the groups. The Mann–Whitney U test results indicated statistically significant differences in Neutral, Happy, and Sad emotional reactions, as evidenced by *p*-values less than 0.05. Effect size calculations (rank biserial correlation) indicated substantial differences for Neutral, Happy, and Sad emotions (*r* = 1.00) but weaker or negligible effects for the other emotions. 

In contrast, the emotional responses of Angry, Surprised, Frightened, Disgusted, Mood State, and Arousal did not show statistically significant differences between the groups. This pattern suggests that while specific emotional responses may differ between groups, this is not uniformly the case across all emotional states. The findings imply that the participants were, in fact, suitable candidates for video self-modeling, as they exhibited significant variations in critical emotional reactions.

### 2.4. Dependent Variables and Response Measurement

The primary purpose of this study was to evaluate the effect of VSM on the acquisition of unplugged coding skills, explicitly using the VEX 123 Robotics Worksheet, in children with ASD. To ensure clarity and consensus regarding the criteria and to facilitate interobserver agreement, the target skill was meticulously detailed in Table 3.

Before the intervention phase, the feasibility of the task analyses for the target skill was assessed using a diverse group of ten individuals differing in age, gender, and educational background. The key metric for this study was the accuracy rate in completing the steps outlined in the task analysis for the VEX 123 Robotics Worksheet. A step was considered correctly executed if a participant independently completed it as specified in Table 2. Conversely, a step was marked as incorrect if the participant failed to perform it as described or did not respond within five seconds.

The percentage of steps completed accurately was calculated by dividing the number of correctly executed steps by the total number of steps in the task analysis and then multiplying this figure by 100% for each participant.

### 2.5. Data Collection and Analysis

This research involved organizing data collection into five main types: effectiveness, maintenance, generalization, social validity, and reliability. Data on effectiveness, maintenance, and generalization were gathered by tracking children’s correct and incorrect answers related to the target skill, allowing for the calculation of correct response percentages. This information was subsequently subjected to graphical analysis to evaluate the intervention’s effects over time and across different contexts and settings. All data were exported in CSV format to enhance statistical analysis and were organized in accordance with single-subject research standards, encompassing session number, phase (baseline/intervention), and response accuracy percentages. The exported files underwent a review process to identify missing values and ensure consistency prior to analysis.

Four statistical effect size measures—Tau-U, Hedges’ g, PAND, and NAP—were meticulously calculated utilizing specialized web-based tools to assess the efficacy of the intervention. These measures were deliberately selected as they address essential limitations inherent in single-subject experimental designs by providing a robust and interpretable evaluation of intervention effects. Single-subject research typically relies on visual analysis; nonetheless, effect size calculations quantify alterations and furnish statistical validation for the findings. Each measure was chosen for its distinct advantages in capturing intervention-related improvements, controlling for baseline trends, and facilitating comparisons with other studies.

The Tau-U value was calculated utilizing the Single-Case Research Tau-U Calculator, recognized for its robust statistical rigor in evaluating effect sizes within single-subject research. In contrast to conventional non-overlap measures, Tau-U effectively integrates both intervention effects and baseline trends, rendering it especially advantageous in scenarios where the baseline data demonstrate variability (Vannest et al., 2016). This methodological approach guarantees that the influence of the intervention is not exaggerated as a consequence of pre-existing trends in the dataset.

Hedges’ g was chosen for its provision of a standardized effect size that is comparable to group-design studies, thereby facilitating meta-analyses and broader research comparisons (Shadish et al., 2016). In contrast to Cohen’s d, which often overestimates effects in small samples, Hedges’ g offers a correction for small-sample bias, rendering it more trustworthy for studies characterized by limited participant numbers, such as single-subject research. The calculation of Hedges’ g involves determining the mean difference between conditions and dividing it by the pooled standard deviation, thus ensuring alignment with established effect size reporting standards.

The Percentage of All Non-Overlapping Data (PAND) has been selected due to its intuitive and visually interpretable characteristics as a measure of effect size. This choice aligns effectively with the graphical analyses commonly utilized in single-subject research (Parker & Vannest, 2009). PAND assesses the proportion of intervention data that surpasses the highest recorded baseline value, thereby providing a clear metric for evaluating treatment efficacy. Furthermore, it can be converted into a Phi coefficient, which serves as a crucial instrument for interpreting the strength of the relationship between various intervention phases.

The Non-Overlap of All Pairs (NAP) has been incorporated as an additional robust non-parametric measure, which is especially advantageous for small sample sizes. NAP evaluates all data points across different phases, rather than merely focusing on baseline and intervention extremes, thus providing a more comprehensive assessment of effect size (Parker & Vannest, 2009). This methodology guarantees that the entirety of the data distribution is taken into account, rather than solely extreme scores, which can occasionally distort findings in research with limited sample sizes.

Each of these effect size measures was computed by employing validated online effect size calculators, thereby ensuring transparency, replicability, and accuracy. The computations provided statistical rigor to supplement the graphical data analysis, thus facilitating a thorough evaluation of the intervention’s impact. Through the incorporation of these measures, this study assures that its findings are both statistically valid and practically significant, consequently reinforcing the role of VSM as an effective instructional tool for fostering unplugged coding skills in children with ASD.

Reliability data, including interobserver agreement and procedural reliability, were gathered from 30% of all experimental sessions. To calculate the interobserver agreement, the point-by-point method outlined by Kazdin (2011) was employed, where the number of agreements was divided by the total number of both agreements and disagreements and then multiplied by 100. These data were collected during each participant’s complete probe, daily probe, intervention, maintenance, and generalization sessions. Procedural reliability was evaluated by comparing the observed behaviors of the researchers to the planned behaviors, following a formula suggested by Kazdin (2011).

Social validity data were also gathered to understand parents’ perspectives on the study. A specially designed questionnaire, comprising six closed-ended and two open-ended questions, was distributed to parents in sealed envelopes. These questions explored various aspects, including the perceived importance and impact of the skills taught, satisfaction with the study, intentions to continue skill practice at home, and openness to future participation in VSM studies. Parents were requested to return their completed questionnaires in sealed envelopes, and their responses were analyzed descriptively.

This comprehensive data collection and analysis approach ensured a thorough evaluation of the intervention’s effectiveness, reliability, and social acceptance, providing a holistic view of its impact on the participants.

*Interobserver Agreement and Procedural Reliability.* This study’s interobserver agreement data showed complete consensus between researchers regarding the participants’ performance across various sessions. This agreement spanned the first and second baseline probe sessions, daily probe sessions, full probe sessions, and maintenance and generalization sessions. Remarkably, the interobserver agreement was calculated to be 100% for all participants, indicating perfect alignment in the researchers’ observations.

The data showed a high level of adherence to the established procedures by the researcher. The procedural reliability was calculated to be 98%, ranging between 99% and 100% for all participants (Cohen’s Kappa: 0.98). This high percentage demonstrates that the researcher consistently and accurately followed the predefined procedures throughout this study, ensuring the reliability and validity of the intervention process.

## 3. Results

### 3.1. Results of Effectiveness, Maintenance, and Generalization

**Figure 1:**
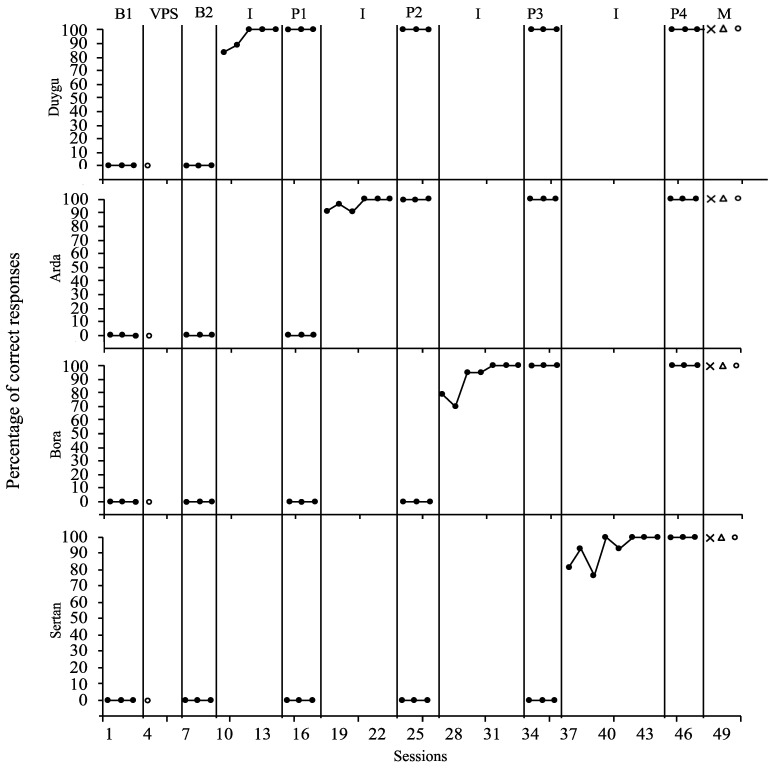
Percentage of correct responses in the baseline, video self-modeling, and follow-up sessions. B: Baseline, VPS: Video preparing session, I: Intervention, P: Probe, ×: first maintenance, 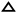: second maintenance, 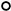: Generalization.

Figure 1 visualizes the effectiveness data, illustrating participants’ performance in the active video game skill across different study phases: baseline, intervention, maintenance, and generalization sessions. In this figure, each data point corresponds to the percentage of correct responses recorded during these sessions.

Figure 1 provides a comprehensive overview of Duygu’s learning of unplugged coding skills. In the first and second baseline probe sessions, Duygu’s performance was at 0%, indicating no initial skill mastery. However, a notable improvement was observed following the introduction of the VSM intervention. Duygu achieved the acquisition criterion, reaching a 100% performance by the third intervention session. This rapid improvement underscores the effectiveness of the VSM approach.

The criteria for concluding the intervention phase were based on maintaining a 100% performance level over three consecutive sessions. Duygu met this criterion, leading to the termination of her intervention sessions after a total of five sessions and ten trials. Her ability to sustain this high level of performance was further demonstrated in all full probe and follow-up sessions, where she consistently showed 100% proficiency. The Tau-U effect size for Duygu was 1.00 (90% CI [−6.36, 8.36]) for the baseline–intervention comparison. A Tau-U effect size of 1.00 indicates 100% non-overlap, demonstrating a substantial improvement. The Hedges’ g value for Duygu was 18.43, representing a very large effect size. The Percentage of Non-Overlapping Data (PAND) was 45.45%, indicating that nearly half of the intervention data points did not overlap with the baseline phase. The Nonoverlap of All Pairs (NAP) value was 1.00, which suggests a complete separation between the baseline and intervention phases, reinforcing the strong effect of the intervention.

**Figure 2:**
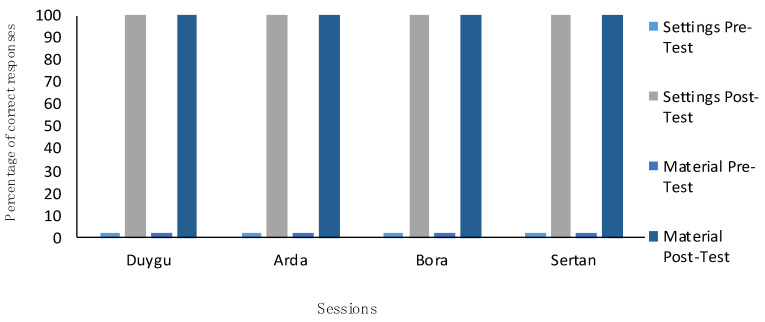
Results of generalization sessions.

Additionally, Figure 2 shows that Duygu’s success extended to the generalization of the skill. She exhibited a 100% performance in the generalization sessions, indicating her ability to apply the skill in different contexts. This consistent performance across various phases of this study highlights the VSM intervention’s effectiveness in teaching and ensuring the skill’s maintenance and generalization.

Figure 1 shows a comprehensive overview of Arda’s learning of unplugged coding skills. In the first and second baseline probe sessions, Arda performed at 0%, indicating no initial skill mastery. However, a notable improvement was observed following the introduction of the VSM intervention. Arda achieved the acquisition criterion, reaching a 100% performance by the third intervention session. This rapid improvement underscores the effectiveness of the VSM approach.

The criteria for concluding the intervention phase were based on maintaining a 100% performance level over three consecutive sessions. Arda met this criterion, leading to the termination of his intervention sessions after a total of five sessions and ten trials. His ability to sustain this high level of performance was further demonstrated in all full probe and follow-up sessions, where he consistently showed 100% proficiency. The Tau-U effect size for Arda was 1.00 (90% CI [−7.39, 9.39]) for the baseline–intervention comparison. This result signifies a 100% non-overlapping effect, demonstrating a substantial improvement. The Hedges’ g value for Arda was 25.45, indicating a powerful effect. The PAND score was 50.00%, meaning that exactly half of the intervention data points were higher than the highest baseline value. The NAP value was 1.00, showing a complete non-overlap between the two phases and suggesting a significant intervention impact.

Additionally, Arda’s success extended to the generalization of the skill. Figure 2 shows that he exhibited a 100% performance level in the generalization sessions, indicating his ability to apply the skill in different contexts. This consistent performance across various phases of this study highlights the VSM intervention’s effectiveness in teaching and ensuring the skill’s maintenance and generalization.

Figure 1 reveals a comprehensive overview of Bora’s learning of unplugged coding skills. In the first and second baseline probe sessions, Bora performed at 0%, indicating no initial skill mastery. However, a notable improvement was observed following the introduction of the VSM intervention. By the third intervention session, Bora achieved the acquisition criterion, reaching a 100% performance level. This rapid improvement underscores the effectiveness of the VSM approach. 

The criteria for concluding the intervention phase were based on maintaining a 100% performance level over three consecutive sessions. Bora met this criterion, leading to the termination of his intervention sessions after a total of five sessions and ten trials. His ability to sustain this high level of performance was further demonstrated in all full probe and follow-up sessions, where he consistently showed 100% proficiency. The Tau-U effect size for Bora was 1.00 (90% CI [−8.40, 10.40]) for the baseline–intervention comparison. This value indicates a 100% non-overlapping intervention effect. The Hedges’ g value for Bora was 9.82, reflecting a large effect size. The PAND result was 53.85%, suggesting that more than half of the intervention data points were higher than the highest baseline value. The NAP value was 1.00, confirming that the intervention separated the baseline and intervention phases.

Additionally, Bora’s success extended to the generalization of the skill. Figure 2 shows that he exhibited a 100% performance level in the generalization sessions, indicating his ability to apply the skill in different contexts. This consistent performance across various phases of this study highlights the VSM intervention’s effectiveness in teaching and ensuring the skill’s maintenance and generalization.

Figure 1 provides a comprehensive overview of Sertan’s learning of unplugged coding skills. In the first and second baseline probe sessions, Sertan performed at 0%, indicating no initial skill mastery. However, a notable improvement was observed following the introduction of the VSM intervention. Sertan achieved the acquisition criterion, reaching a 100% performance by the third intervention session. This rapid improvement underscores the effectiveness of the VSM approach.

The criteria for concluding the intervention phase were based on maintaining a 100% performance level over three consecutive sessions. Sertan met this criterion, leading to the termination of his intervention sessions after a total of five sessions and ten trials. His ability to sustain this high level of performance was further demonstrated in all full probe and follow-up sessions, where he consistently showed 100% proficiency. The Tau-U effect size for Sertan was 1.00 (90% CI [−9.40, 11.40]) for the baseline–intervention comparison, demonstrating a 100% non-overlapping effect. The Hedges’ g value for Sertan was 11.72, suggesting a significant intervention effect. The PAND score was 57.14%, meaning most intervention data points exceeded the baseline values. The NAP value was 1.00, indicating a complete non-overlap between the baseline and intervention phases, reinforcing the effectiveness of the intervention.

Additionally, Sertan’s success extended to the generalization of the skill. Figure 2 shows that he exhibited a 100% performance level in the generalization sessions, indicating his ability to apply the skill in different contexts. This consistent performance across various phases of this study highlights the VSM intervention’s effectiveness in teaching and ensuring the skill’s maintenance and generalization.

#### Results of Social Validity

The social validity questionnaire forms, administered face-to-face and collected from parents after the intervention process, played a crucial role in assessing the perceived value and impact of this study from the parents’ perspective. The questionnaire comprised six closed-ended and two open-ended questions designed to gather comprehensive feedback on various aspects of this study.

As shown in Table 4, in response to the first closed-ended question, all parents unanimously agreed that learning unplugged coding skills was necessary for their child. This consensus highlights the perceived relevance and significance of the skills taught, suggesting that parents saw them as a valuable addition to their child’s abilities. Regarding the second closed-ended question, all parents acknowledged that unplugged coding skills would contribute to their child’s repertoire of STEM skills. This response indicates that parents viewed the skill as an educational or therapeutic tool to enrich their child’s repertoire. It suggests that they recognized the importance of such skills in providing a well-rounded and enjoyable experience for their children, especially considering the need for engaging and appropriate activities for children with specific needs. These responses from the parents provide valuable insights into the social validity of this study, affirming that the skills taught were not only practical in a controlled setting but also valued and applicable in the everyday lives of the participants and their parents.

## 4. Discussion

This study aimed to evaluate the effectiveness of VSM in teaching unplugged coding skills to children diagnosed with ASD. The results demonstrate that VSM was an effective method for imparting unplugged coding skills to four children with ASD who participated. A consistent pattern of effectiveness was observed across all participants, with the acquisition of the targeted skills typically occurring within five to six sessions. One exception was noted in the case of a participant named Sertan, who reached the desired skill level after eight sessions. These findings underscore VSM’s potential as a structured, engaging, and effective approach to introducing computational thinking skills to children with ASD.

This is the first study to use VSM to teach STEM skills, specifically unplugged coding skills. This study focused on non-diagnostic skills, such as coding, due to the limited range of STEM skills and participation in STEM-related activities among children with ASD (Little et al., 2014). Therefore, the results contribute to the literature by demonstrating the effectiveness of VSM in teaching children with ASD the foundational skills necessary for coding activities in school or other settings, as well as enhancing their participation in STEM education. Given that STEM-related problem-solving, logical reasoning, and computational thinking skills are essential for future academic and career opportunities, this study provides a valuable framework for incorporating ASD-friendly teaching methodologies into STEM education.

Upon reviewing VSM research, numerous studies have utilized multiple baseline designs (e.g., Bellini et al., 2007; Boudreau & Harvey, 2013; Buggey, 2005b, 2012; Cihak & Schrader, 2008; Delano, 2007; Hart & Whalon, 2012; Lang et al., 2009; Litras et al., 2010; Nikopoulos & Panagiotopoulou, 2015; Wert & Neisworth, 2003; Williamson et al., 2013) and comparison designs (Marcus & Wilder, 2009; Sherer et al., 2001) as their experimental frameworks. However, these studies generally implemented a single baseline probe condition. This singular approach may have drawbacks, particularly regarding the creation of video clips, which can inadvertently lead to incidental learning among participants and thus affect intervention session data. This occurs because video recordings showcase a step-by-step skill demonstration during the production of VSM clips. To mitigate this issue, it is advisable to incorporate probe conditions both before and after video production. Several studies, such as Goh and Bambara (2013), Litras et al. (2010), Nikopoulos and Panagiotopoulou (2015), and Kurnaz and Yanardağ (2018), have utilized this method by implementing two baseline probes prior to intervention. In the current study, two baseline probes were conducted before and after the video recording sessions to identify potential changes in the dependent variable. A second baseline assessment was conducted to reduce the chance of incidental learning after the video clip session. The initial data points from this second baseline matched those of the first, confirming the absence of incidental learning, and following that, VSM intervention sessions were delivered. No incidental learning was observed after video production, aligning with the findings from previous studies. The rapid mastery of unplugged coding skills noted in this study correlates with prior research highlighting the benefits of VSM (Bellini et al., 2007; Litras et al., 2010). The positive learning outcomes observed indicate that self-modeling may enhance engagement, motivation, and self-efficacy in children with ASD, especially in structured educational interventions. Thus, the current study’s methodological design offers a novel contribution to the existing body of literature.

The rationale for employing VSM with children diagnosed with ASD is based on the assumption that these individuals show reduced reactivity to their own images compared to their reactions when watching videos of others. Previous research has indicated that individuals with autism may respond differently to visual stimuli and may exhibit a greater preference for observing their own images (Buggey, 2005a; Keenan & Nikopoulos, 2006). However, empirical investigations directly validating this hypothesis have been limited. This study makes a significant contribution to the existing literature as it is the first effort to confirm, through Noldus FaceReader analysis, the idea that children with autism prefer viewing their own videos over other visual stimuli. The findings from the Noldus FaceReader analysis indicated that while engaged with their own videos, the children showed more neutral and positive emotional responses compared to the more negative reactions observed during the viewing of other videos. This result suggests that VSM might serve as a more effective and acceptable educational approach for children with autism. Furthermore, it underscores the potential of VSM in improving self-regulation skills among this population. Future research is encouraged to incorporate similar analytical techniques and to design studies aimed at further exploring the effectiveness of VSM as well as the emotional responses of individuals with autism to their personalized visual stimuli. This approach will enable a more systematic assessment of the long-term effects of VSM on learning motivation, social interactions, and cognitive engagement.

The findings of this study support previous research using VSM to teach various chaining skills to children with ASD. Studies have shown that VSM enhances spontaneous requests and initiates peer interactions (Marcus & Wilder, 2009) and improves complex verbal skills such as conversational speech (Charlop & Milstein, 1989). VSM has also proven particularly effective in addressing behavioral challenges and fostering cognitive engagement in individuals with ASD (Frolli et al., 2020).

Empirical evidence also supports VSM’s role in enhancing language and communication abilities, social skills, behavior modification, and functional skill development (Gelbar et al., 2012). It has been utilized to teach self-help skills to children with disabilities (Norman et al., 2001) and continues to be effective for various social, communicative, functional, behavioral, play, and self-help skills in students with ASD (Hughes & Yakubova, 2016).

While both robotic coding and unplugged coding activities have gained recognition for their potential to enhance cognitive and social skills in children with ASD (Smith, 2018), unplugged coding, which teaches coding concepts without the use of computers, provides a low-stimulation, structured learning environment that may better align with the learning profiles of children with ASD (Knight et al., 2019b). Incorporating VSM into unplugged coding instruction could serve as an innovative way to introduce computational thinking skills while minimizing sensory overload and maximizing engagement.

Based on feedback regarding social validity from the parents of participants, there was a consensus on the positive perception of the intervention’s approach, goals, and outcomes, particularly in fostering autonomy and enhancing skills in STEM and coding. Parents indicated that their children continued to engage with unplugged coding activities after the intervention, suggesting that VSM had a lasting influence on their interest and involvement in computational thinking tasks.

A review of the existing literature indicates that only a limited number of studies (Bellini et al., 2007; Cihak & Schrader, 2008; Boudreau & Harvey, 2013; Kurnaz & Yanardağ, 2018) have included social validity data in their applications of VSM. Consequently, this study enhances the current body of social validity data relevant to the VSM methodology, providing significant insights into its practical feasibility and acceptance among families. While social validity data confirm the intervention’s acceptability and perceived effectiveness, it is important to recognize the challenges associated with real-world implementation. A common issue is the time-intensive nature of VSM preparation, as video production requires careful scripting, recording, and editing to ensure clarity and effectiveness. For educators and therapists working with larger groups of children, this factor may pose a scalability challenge. One potential solution is to create premade, adaptable VSM templates that can be easily modified for individual learners, reducing the time burden on practitioners.

Another challenge is ensuring that learned skills generalize beyond the structured intervention setting. While parents reported that their children remained interested in coding activities, future studies should investigate how VSM-based learning translates into real-world problem-solving and academic contexts. One strategy to address this issue is to embed VSM interventions within classroom activities or combine them with hands-on coding exercises to enhance skill transfer and retention. Moreover, parental involvement is vital for maintaining the effectiveness of interventions. Although this study successfully collected parental feedback, formal training programs for parents on using VSM at home could further improve its long-term effects. Providing accessible guides or video tutorials on creating and implementing VSM clips can empower parents to incorporate these strategies into their daily routines.

A review of previous VSM studies shows that only a limited number (Bellini et al., 2007; Cihak & Schrader, 2008; Boudreau & Harvey, 2013; Kurnaz & Yanardağ, 2018) have included detailed social validity assessments. This study builds on existing work by highlighting both the benefits and challenges of VSM implementation in STEM education. Future research should explore additional stakeholder perspectives, including teachers and therapists, to gain a more comprehensive understanding of VSM’s feasibility across various learning environments. This study addresses these challenges and explores practical strategies for overcoming them, contributing valuable insights for educators, researchers, and practitioners seeking to implement VSM as an evidence-based intervention for STEM learning in children with ASD.

This study has certain limitations. First, the sample size was restricted to just four children with ASD, limiting the findings’ generalizability. Additionally, the research concentrated on a single skill as the dependent variable. The time-consuming process of creating the VSM clips emerged as another limitation. Future research could address this by simplifying the technology and editing software used (Schaeffer et al., 2016).

Additionally, the evaluation of participants’ skill sets was not performed systematically, indicating that future research could benefit from structured assessment tools to accurately evaluate individual skill levels both prior to and following the intervention. Additionally, upcoming studies should broaden their focus to include a larger and more diverse participant demographic, as well as a wider range of unplugged coding skills, aiming to further promote the application of STEM skills among children diagnosed with ASD.

Additionally, alternative video-based instructional methods, such as video modeling, should be compared with VSM to evaluate their effectiveness and efficiency. A notable research gap exists regarding the impact of VSM on various coding skills in children with ASD. Future studies should also assess engagement levels in coding and STEM activities both before and after the intervention to determine long-term effects.

## Figures and Tables

**Table 1:** Participant characteristics.

Name	Gender	Age	Diagnosis	GARS-2-TV
Duygu	Female	10	ASD	77
Arda	Male	10	ASD	79
Bora	Male	12	ASD	79
Sertan	Male	12	ASD	75

Note. ASD = Autism spectrum disorder; GARS-2-TV = The Gilliam Autism Rating Scale-2-Turkish Version.

**Table 2:** Results of analyses of participants’ emotional expressions when viewing their images.

Groups	Self Video			Blank Video					
Emotions	N	Mean Rank	Sum of Ranks	N	Mean Rank	Sum of Ranks	U	p	r
Neutral	4	2.50	10.00	4	6.50	26.00	0.000	0.021	1.0
Happy	4	6.50	26.00	4	2.50	10.00	0.000	0.021	1.0
Sad	4	2.50	10.00	4	6.50	26.00	0.000	0.021	1.0
Angry	4	4.25	17.00	4	4.75	19.00	7.000	0.773	0.125
Surprised	4	5.50	22.00	4	3.50	14.00	4.000	0.248	0.5
Scared	4	3.75	15.00	4	5.25	21.00	5.000	0.386	0.375
Disgusted	4	4.00	16.00	4	5.00	20.00	6.000	0.564	0.25
Valence	4	6.00	24.00	4	3.00	12.00	2.000	0.083	0.75
Arousal	4	5.25	21.00	4	3.75	15.00	5.000	0.386	0.375

**Table 3:** Coding system.

Behaviors	Score
Goes from the green circle to the end point.	5
Draws the directional arrows from the circle to the point of the green circle.	4
Draws the arrows from the point of the red circle to the point of the yellow circle.	3
Draws the directional arrows from the start point to the red circle.	2
Sets the starting point	1
No response	0

**Table 4:** Results of social validity.

Questions	Yes	No	Undecided
Do you think the researcher fulfilled his/her responsibilities specified in the contract in this research?	4	—	—
Is it important for you that your student learns the use of unplugged coding skills?	4	—	—
Are you satisfied with the use of video self-modeling teaching process in teaching unplugged coding skills?	4	—	—
Is video self-modeling an appropriate method for teaching the use of unplugged coding skills?	4	—	—
Is it easy for you to use the video self-modelling teaching process?	4	—	—

Note. The second column indicates the number of participants who responded to each question. No participants provided any responses.

## Data Availability

Data are unavailable due to privacy or ethical restrictions approved by Anadolu University.
